# Navigating Neurocognitive Territory: Late-Onset Bipolar Disorder Insights

**DOI:** 10.1192/j.eurpsy.2024.1325

**Published:** 2024-08-27

**Authors:** D. V. Cotovio, F. Agostinho, M. R. Resende, R. S. Lousada, R. S. Nogueira, F. A. Silva, M. M. Melo

**Affiliations:** Departamento de Psiquiatria e Saúde Mental, Hospital Beatriz Ângelo, Loures, Portugal

## Abstract

**Introduction:**

Affective disorders are associated with cognitive deterioration, manifested by an increased risk of developing dementia. Late-onset bipolar disorder (BD) establishes a dynamic interaction between dementia and BD, considering its particular manifestations in old age.

**Objectives:**

Provide a comprehensive overview of the clinical and epidemiological attributes specific to late-onset BD, elucidating its interplay with dementia.

**Methods:**

We conducted a literature search on PubMed in August 2023, using the following terms: late-onset bipolar disorder AND dementia. Only systematic reviews and meta-analysis were included with no year or language restrictions. Three articles were eligible for this review: two systematic reviews and one meta-analysis.

**Results:**

Late-onset BD can be defined as a secondary condition and may result from an expression of lower vulnerability to BD, when compared to early-onset BD. On the other hand, late-onset BD may be conceptualized as a subtype of pseudodementia, or even considered a risk factor for dementia. In fact, this particular association with dementia supports the existence of a specific class of BD, i.e. BD type VI. Such diagnostic overlap might be explained by common factors that have been associated with both BD and dementia, such as cardiovascular risk factors, systemic inflammation, stress and levels of baseline cognitive reserve. Despite the commonalities, other aspects, such as family history and prior history of a mood disorder, may help to make the differential diagnosis between late-onset BD and dementia.

**Conclusions:**

There is a diagnostic challenge between dementia and the neurocognitive decline associated with BD, particularly in the case of a late-onset BD. Although the available evidence is limited, current evidence demonstrates that BD can indeed be seen as a risk factor for dementia. Therefore, cognitive impairment in individuals with BD should not be overlooked.

**Disclosure of Interest:**

None Declared

